# Death of Mr. Joberns

**Published:** 1833-01

**Authors:** 


Death of Mr. Joberns. Mr. Joberns, for many years senior surgeon of the Middlesex Hospital, and inspector general of hospitals, died recently at Woodstock. Mr. Joberns' health had for some time been declining, and it was supposed by his professional friends that he was labouring under organic disease of the heart, and this opinion is strengthened by his sudden and unexpected death. We are intimately acquainted with many persons who long knew Mr. Joberns, and we have invariably heard him spoken of in terms of esteem and respect. As a surgeon, he had not, we believe, distinguished himself by any splendid or original contributions to our stock of knowledge; but he was nevertheless an able practitioner, and very fertile in medical resources when others had exhausted their means of relief. For some years before his death, he performed no surgical operations, but his opinion in cases of danger and doubt was always received by his colleagues with the respect and attention to which his long experience and great practical knowledge justly entitled him.

Our regret for the loss of Mr. Joberns is fortunately not increased by any doubt of the entire competency of the gentleman who will now have to perform the responsible duties of surgeon to the Middlesex hospital; for Mr. Arnott, at present assistant surgeon to the hospital, will doubtless become the surgeon without contest; but for the *probable* vacancy in the assistant surgeoncy there are three candidates in the field, namely, Mr. Alexander Shaw, Mr. Tuson, and Mr. Phillips.

